# Supplementary material for: Human brain-wide activation of sleep rhythms
Source: eLife. 2026 Aug 12;14:RP103956. doi: 10.7554/eLife.103956 (PMC13466540; doi:10.7554/eLife.103956)
Supplement: Supplementary file 2 [file elife-103956-supp1.docx]

| **Descriptive statistics of demographic and sleep** | **Mean ± SE** |
| --- | --- |
|  | **(n = 107)** |
| Age | 22.40 ± 0.34 |
| Total Recording time (min) | 180.60 ± 8.40 |
| Wake time (min) | 84.60 ± 5.00 |
| Total Sleep time (min) | 95.98 ± 5.67 |
| N1 time (min) | 13.99 ± 1.19 |
| N2 time (min) | 56.21 ± 4.05 |
| N3 time (min) | 16.53 ± 1.35 |
| REM time (min) | 9.27 ± 0.86 |
| N1 ratio (%) | 14.57 ± 1.06 |
| N2 ratio (%) | 58.55 ± 1.70 |
| N3 ratio (%) | 17.22 ± 1.21 |
| REM ratio (%) | 9.66 ± 0.90 |
| Sleep Onset Latency (min) | 9.75 ± 0.85 |
| N2 latency (min) | 14.58 ± 1.15 |
| N3 latency (min) | 33.65 ± 3.02 |
| Sleep Efficiency (%) | 52.96 ± 1.60 |

**Table S1 | Descriptive results of demographic information and sleep characteristics**. Note: The total recorded time is equal to the awake time plus the total sleep time. The sleep onset latency is the time taken to reach the first sleep epoch. The Sleep Efficiency is the ratio of actual sleep time to total recording time.
