# Supplementary material for: Human brain-wide activation of sleep rhythms
Source: eLife. 2026 Aug 12;14:RP103956. doi: 10.7554/eLife.103956 (PMC13466540; doi:10.7554/eLife.103956)
Supplement: Supplementary file 3 [file elife-103956-supp2.docx]

| **Subject ID** | **N1 Sleep Stage** | | | | | | |
| --- | --- | --- | --- | --- | --- | --- | --- |
|  | **Time (min)** | **Number of SO** | **Number of spindle** | **Number of coupling** | **Density of SO (times/min)** | **Density of spindle (times/min)** | **Density of coupling (times/min)** |
| 1 | 8.38 | 27 | 24 | 6 | 3.22 | 2.87 | 0.72 |
| 2 | 5.21 | 26 | 23 | 8 | 4.99 | 4.41 | 1.54 |
| 3 | 3.24 | 14 | 14 | 4 | 4.32 | 4.32 | 1.23 |
| 4 | 16.61 | 25 | 49 | 6 | 1.51 | 2.95 | 0.36 |
| 5 | 3.38 | 0 | 0 | 0 | 0.00 | 0.00 | 0.00 |
| 6 | 9.74 | 35 | 34 | 11 | 3.59 | 3.49 | 1.13 |
| 7 | 8.84 | 25 | 16 | 11 | 2.83 | 1.81 | 1.24 |
| 8 | 19.51 | 96 | 116 | 40 | 4.92 | 5.95 | 2.05 |
| 9 | 2.28 | 5 | 2 | 1 | 2.20 | 0.88 | 0.44 |
| 10 | 25.71 | 29 | 11 | 3 | 1.13 | 0.43 | 0.12 |
| 11 | 8.04 | 24 | 19 | 5 | 2.98 | 2.36 | 0.62 |
| 12 | 4.54 | 6 | 5 | 1 | 1.32 | 1.10 | 0.22 |
| 13 | 4.91 | 15 | 22 | 5 | 3.05 | 4.48 | 1.02 |
| 14 | 3.41 | 3 | 15 | 1 | 0.88 | 4.40 | 0.29 |
| 15 | 4.91 | 10 | 9 | 2 | 2.04 | 1.83 | 0.41 |
| 16 | 21.98 | 39 | 113 | 14 | 1.77 | 5.14 | 0.64 |
| 17 | 1.31 | 8 | 7 | 4 | 6.11 | 5.34 | 3.05 |
| 18 | 0.91 | 0 | 0 | 0 | 0.00 | 0.00 | 0.00 |
| 19 | 2.54 | 10 | 8 | 4 | 3.93 | 3.15 | 1.57 |
| 20 | 23.71 | 78 | 64 | 23 | 3.29 | 2.70 | 0.97 |
| 21 | 35.81 | 72 | 50 | 13 | 2.01 | 1.40 | 0.36 |
| 22 | 25.58 | 42 | 103 | 20 | 1.64 | 4.03 | 0.78 |
| 23 | 3.41 | 0 | 0 | 0 | 0.00 | 0.00 | 0.00 |
| 24 | 20.68 | 48 | 50 | 13 | 2.32 | 2.42 | 0.63 |
| 25 | 47.74 | 142 | 192 | 51 | 2.97 | 4.02 | 1.07 |
| 26 | 20.91 | 66 | 43 | 16 | 3.16 | 2.06 | 0.77 |
| 27 | 7.91 | 48 | 38 | 12 | 6.07 | 4.80 | 1.52 |
| 28 | 10.71 | 30 | 42 | 12 | 2.80 | 3.92 | 1.12 |
| 29 | 22.71 | 99 | 74 | 31 | 4.36 | 3.26 | 1.37 |
| 30 | 2.84 | 14 | 12 | 2 | 4.92 | 4.22 | 0.70 |
| 31 | 7.41 | 17 | 6 | 2 | 2.29 | 0.81 | 0.27 |
| 32 | 0.98 | 0 | 0 | 0 | 0.00 | 0.00 | 0.00 |
| 33 | 3.21 | 20 | 12 | 4 | 6.23 | 3.74 | 1.25 |
| 34 | 7.81 | 32 | 13 | 4 | 4.10 | 1.66 | 0.51 |
| 35 | 4.44 | 14 | 6 | 2 | 3.15 | 1.35 | 0.45 |
| 36 | 41.34 | 123 | 101 | 28 | 2.98 | 2.44 | 0.68 |
| 37 | 5.31 | 27 | 17 | 12 | 5.08 | 3.20 | 2.26 |
| 38 | 13.34 | 39 | 38 | 13 | 2.92 | 2.85 | 0.97 |
| 39 | 20.34 | 64 | 36 | 8 | 3.15 | 1.77 | 0.39 |
| 40 | 5.74 | 28 | 16 | 6 | 4.88 | 2.79 | 1.04 |
| 41 | 8.11 | 1 | 16 | 1 | 0.12 | 1.97 | 0.12 |
| 42 | 25.68 | 63 | 105 | 17 | 2.45 | 4.09 | 0.66 |
| 43 | 23.21 | 46 | 17 | 9 | 1.98 | 0.73 | 0.39 |
| 44 | 16.94 | 44 | 66 | 10 | 2.60 | 3.90 | 0.59 |
| 45 | 6.31 | 11 | 20 | 3 | 1.74 | 3.17 | 0.48 |
| 46 | 7.24 | 41 | 20 | 5 | 5.66 | 2.76 | 0.69 |
| 47 | 9.14 | 32 | 27 | 11 | 3.50 | 2.95 | 1.20 |
| 48 | 35.71 | 139 | 177 | 54 | 3.89 | 4.96 | 1.51 |
| 49 | 7.01 | 15 | 27 | 7 | 2.14 | 3.85 | 1.00 |
| 50 | 27.18 | 117 | 55 | 18 | 4.31 | 2.02 | 0.66 |
| 51 | 13.38 | 42 | 53 | 13 | 3.14 | 3.96 | 0.97 |
| 52 | 17.54 | 48 | 42 | 4 | 2.74 | 2.39 | 0.23 |
| 53 | 24.31 | 140 | 78 | 30 | 5.76 | 3.21 | 1.23 |
| 54 | 11.54 | 37 | 60 | 13 | 3.21 | 5.20 | 1.13 |
| 55 | 10.14 | 2 | 27 | 1 | 0.20 | 2.66 | 0.10 |
| 56 | 4.41 | 27 | 21 | 9 | 6.12 | 4.76 | 2.04 |
| 57 | 5.01 | 19 | 3 | 1 | 3.79 | 0.60 | 0.20 |
| 58 | 6.01 | 28 | 28 | 7 | 4.66 | 4.66 | 1.16 |
| 59 | 37.38 | 136 | 93 | 30 | 3.64 | 2.49 | 0.80 |
| 60 | 7.08 | 35 | 9 | 3 | 4.95 | 1.27 | 0.42 |
| 61 | 8.74 | 38 | 36 | 9 | 4.35 | 4.12 | 1.03 |
| 62 | 17.61 | 45 | 24 | 12 | 2.56 | 1.36 | 0.68 |
| 63 | 48.61 | 125 | 148 | 31 | 2.57 | 3.04 | 0.64 |
| 64 | 9.74 | 29 | 54 | 10 | 2.98 | 5.54 | 1.03 |
| 65 | 22.21 | 40 | 47 | 9 | 1.80 | 2.12 | 0.41 |
| 66 | 36.78 | 63 | 52 | 14 | 1.71 | 1.41 | 0.38 |
| 67 | 33.51 | 37 | 54 | 13 | 1.10 | 1.61 | 0.39 |
| 68 | 10.71 | 50 | 11 | 3 | 4.67 | 1.03 | 0.28 |
| 69 | 58.34 | 143 | 130 | 31 | 2.45 | 2.23 | 0.53 |
| 70 | 14.21 | 50 | 55 | 16 | 3.52 | 3.87 | 1.13 |
| 71 | 21.98 | 88 | 74 | 26 | 4.00 | 3.37 | 1.18 |
| 72 | 55.31 | 212 | 203 | 45 | 3.83 | 3.67 | 0.81 |
| 73 | 24.61 | 51 | 19 | 5 | 2.07 | 0.77 | 0.20 |
| 74 | 23.31 | 100 | 47 | 27 | 4.29 | 2.02 | 1.16 |
| 75 | 4.74 | 10 | 7 | 2 | 2.11 | 1.48 | 0.42 |
| 76 | 37.11 | 77 | 34 | 9 | 2.07 | 0.92 | 0.24 |
| 77 | 14.41 | 55 | 25 | 12 | 3.82 | 1.73 | 0.83 |
| 78 | 12.21 | 0 | 0 | 0 | 0.00 | 0.00 | 0.00 |
| 79 | 6.18 | 0 | 0 | 0 | 0.00 | 0.00 | 0.00 |
| 80 | 8.18 | 2 | 16 | 1 | 0.24 | 1.96 | 0.12 |
| 81 | 2.84 | 9 | 5 | 2 | 3.17 | 1.76 | 0.70 |
| 82 | 7.41 | 19 | 21 | 6 | 2.56 | 2.83 | 0.81 |
| 83 | 1.91 | 11 | 5 | 2 | 5.76 | 2.62 | 1.05 |
| 84 | 2.14 | 11 | 4 | 1 | 5.13 | 1.87 | 0.47 |
| 85 | 10.38 | 24 | 38 | 7 | 2.31 | 3.66 | 0.67 |
| 86 | 10.34 | 43 | 55 | 14 | 4.16 | 5.32 | 1.35 |
| 87 | 5.98 | 20 | 16 | 3 | 3.35 | 2.68 | 0.50 |
| 88 | 0.41 | 0 | 0 | 0 | 0.00 | 0.00 | 0.00 |
| 89 | 10.91 | 16 | 48 | 5 | 1.47 | 4.40 | 0.46 |
| 90 | 9.54 | 45 | 28 | 11 | 4.72 | 2.93 | 1.15 |
| 91 | 4.18 | 26 | 11 | 3 | 6.23 | 2.63 | 0.72 |
| 92 | 2.14 | 0 | 0 | 0 | 0.00 | 0.00 | 0.00 |
| 93 | 10.11 | 26 | 35 | 9 | 2.57 | 3.46 | 0.89 |
| 94 | 1.91 | 3 | 10 | 2 | 1.57 | 5.24 | 1.05 |
| 95 | 8.68 | 11 | 24 | 4 | 1.27 | 2.77 | 0.46 |
| 96 | 30.71 | 112 | 99 | 28 | 3.65 | 3.22 | 0.91 |
| 97 | 11.71 | 22 | 15 | 5 | 1.88 | 1.28 | 0.43 |
| 98 | 11.91 | 42 | 31 | 8 | 3.53 | 2.60 | 0.67 |
| 99 | 16.41 | 42 | 69 | 15 | 2.56 | 4.20 | 0.91 |
| 100 | 7.34 | 23 | 27 | 5 | 3.13 | 3.68 | 0.68 |
| 101 | 6.41 | 13 | 18 | 3 | 2.03 | 2.81 | 0.47 |
| 102 | 9.24 | 33 | 28 | 7 | 3.57 | 3.03 | 0.76 |
| 103 | 3.34 | 20 | 12 | 5 | 5.98 | 3.59 | 1.50 |
| 104 | 13.41 | 30 | 66 | 9 | 2.24 | 4.92 | 0.67 |
| 105 | 10.58 | 5 | 26 | 3 | 0.47 | 2.46 | 0.28 |
| 106 | 6.48 | 22 | 20 | 9 | 3.40 | 3.09 | 1.39 |
| 107 | 21.51 | 65 | 6 | 2 | 3.02 | 0.28 | 0.09 |
| **Mean** | 13.99 | 40.48 | 38.29 | 10.16 | 2.95 | 2.71 | 0.75 |
| **SE** | 1.19 | 3.85 | 3.92 | 1.06 | 0.16 | 0.14 | 0.05 |

**Table S2 | Statistics of sleep duration, SO, spindle and coupling event numbers and densities for all 107 subjects during N1 stage.**
