# Supplementary material for: Human brain-wide activation of sleep rhythms
Source: eLife. 2026 Aug 12;14:RP103956. doi: 10.7554/eLife.103956 (PMC13466540; doi:10.7554/eLife.103956)
Supplement: Supplementary file 4 [file elife-103956-supp3.docx]

| **Subject ID** | **N2&N3 Sleep Stage** | | | | | | |
| --- | --- | --- | --- | --- | --- | --- | --- |
|  | **Time (min)** | **Number of SO** | **Number of spindle** | **Number of coupling** | **Density of SO (times/min)** | **Density of spindle (times/min)** | **Density of coupling (times/min)** |
| 1 | 89.00 | 820 | 401 | 242 | 9.21 | 4.51 | 2.72 |
| 2 | 125.00 | 1417 | 659 | 298 | 11.34 | 5.27 | 2.38 |
| 3 | 22.50 | 225 | 115 | 75 | 10.00 | 5.11 | 3.33 |
| 4 | 87.00 | 1059 | 326 | 175 | 12.17 | 3.75 | 2.01 |
| 5 | 44.50 | 425 | 190 | 115 | 9.55 | 4.27 | 2.58 |
| 6 | 21.00 | 222 | 78 | 57 | 10.57 | 3.71 | 2.71 |
| 7 | 42.50 | 185 | 160 | 91 | 4.35 | 3.76 | 2.14 |
| 8 | 167.50 | 1865 | 882 | 502 | 11.13 | 5.27 | 3.00 |
| 9 | 25.50 | 112 | 28 | 24 | 4.39 | 1.10 | 0.94 |
| 10 | 65.00 | 715 | 322 | 197 | 11.00 | 4.95 | 3.03 |
| 11 | 69.00 | 511 | 302 | 155 | 7.41 | 4.38 | 2.25 |
| 12 | 62.50 | 413 | 316 | 162 | 6.61 | 5.06 | 2.59 |
| 13 | 89.00 | 826 | 449 | 283 | 9.28 | 5.04 | 3.18 |
| 14 | 41.00 | 390 | 191 | 93 | 9.51 | 4.66 | 2.27 |
| 15 | 105.00 | 359 | 96 | 51 | 3.42 | 0.91 | 0.49 |
| 16 | 184.50 | 708 | 425 | 228 | 3.84 | 2.30 | 1.24 |
| 17 | 97.50 | 395 | 306 | 155 | 4.05 | 3.14 | 1.59 |
| 18 | 23.50 | 212 | 86 | 75 | 9.02 | 3.66 | 3.19 |
| 19 | 17.50 | 137 | 48 | 39 | 7.83 | 2.74 | 2.23 |
| 20 | 102.00 | 1196 | 462 | 290 | 11.73 | 4.53 | 2.84 |
| 21 | 139.00 | 1236 | 671 | 359 | 8.89 | 4.83 | 2.58 |
| 22 | 56.50 | 684 | 284 | 166 | 12.11 | 5.03 | 2.94 |
| 23 | 43.00 | 508 | 182 | 117 | 11.81 | 4.23 | 2.72 |
| 24 | 75.50 | 872 | 313 | 194 | 11.55 | 4.15 | 2.57 |
| 25 | 136.00 | 1616 | 792 | 408 | 11.88 | 5.82 | 3.00 |
| 26 | 29.00 | 190 | 118 | 63 | 6.55 | 4.07 | 2.17 |
| 27 | 73.50 | 849 | 408 | 229 | 11.55 | 5.55 | 3.12 |
| 28 | 249.50 | 2355 | 1170 | 806 | 9.44 | 4.69 | 3.23 |
| 29 | 54.00 | 603 | 224 | 137 | 11.17 | 4.15 | 2.54 |
| 30 | 7.50 | 34 | 16 | 13 | 4.53 | 2.13 | 1.73 |
| 31 | 30.50 | 269 | 139 | 91 | 8.82 | 4.56 | 2.98 |
| 32 | 53.00 | 550 | 258 | 128 | 10.38 | 4.87 | 2.42 |
| 33 | 24.00 | 290 | 128 | 68 | 12.08 | 5.33 | 2.83 |
| 34 | 44.00 | 401 | 182 | 111 | 9.11 | 4.14 | 2.52 |
| 35 | 14.00 | 120 | 62 | 36 | 8.57 | 4.43 | 2.57 |
| 36 | 177.50 | 1925 | 782 | 518 | 10.85 | 4.41 | 2.92 |
| 37 | 113.50 | 1290 | 569 | 345 | 11.37 | 5.01 | 3.04 |
| 38 | 113.50 | 1286 | 570 | 303 | 11.33 | 5.02 | 2.67 |
| 39 | 72.50 | 660 | 230 | 134 | 9.10 | 3.17 | 1.85 |
| 40 | 119.00 | 827 | 284 | 176 | 6.95 | 2.39 | 1.48 |
| 41 | 43.00 | 438 | 176 | 101 | 10.19 | 4.09 | 2.35 |
| 42 | 79.50 | 917 | 324 | 154 | 11.53 | 4.08 | 1.94 |
| 43 | 197.00 | 1166 | 559 | 369 | 5.92 | 2.84 | 1.87 |
| 44 | 93.00 | 1136 | 362 | 205 | 12.22 | 3.89 | 2.20 |
| 45 | 159.00 | 696 | 411 | 227 | 4.38 | 2.58 | 1.43 |
| 46 | 53.00 | 575 | 165 | 102 | 10.85 | 3.11 | 1.92 |
| 47 | 143.00 | 596 | 317 | 207 | 4.17 | 2.22 | 1.45 |
| 48 | 149.00 | 1514 | 850 | 515 | 10.16 | 5.70 | 3.46 |
| 49 | 27.00 | 312 | 102 | 61 | 11.56 | 3.78 | 2.26 |
| 50 | 110.00 | 707 | 339 | 238 | 6.43 | 3.08 | 2.16 |
| 51 | 42.50 | 439 | 144 | 71 | 10.33 | 3.39 | 1.67 |
| 52 | 52.00 | 275 | 171 | 74 | 5.29 | 3.29 | 1.42 |
| 53 | 34.00 | 343 | 136 | 91 | 10.09 | 4.00 | 2.68 |
| 54 | 69.00 | 820 | 359 | 198 | 11.88 | 5.20 | 2.87 |
| 55 | 81.00 | 940 | 327 | 197 | 11.60 | 4.04 | 2.43 |
| 56 | 39.50 | 448 | 209 | 124 | 11.34 | 5.29 | 3.14 |
| 57 | 32.50 | 301 | 140 | 76 | 9.26 | 4.31 | 2.34 |
| 58 | 51.50 | 525 | 218 | 119 | 10.19 | 4.23 | 2.31 |
| 59 | 55.50 | 561 | 212 | 150 | 10.11 | 3.82 | 2.70 |
| 60 | 114.00 | 1188 | 485 | 279 | 10.42 | 4.25 | 2.45 |
| 61 | 59.50 | 713 | 373 | 194 | 11.98 | 6.27 | 3.26 |
| 62 | 70.50 | 795 | 250 | 204 | 11.28 | 3.55 | 2.89 |
| 63 | 105.00 | 693 | 432 | 231 | 6.60 | 4.11 | 2.20 |
| 64 | 57.00 | 653 | 347 | 172 | 11.46 | 6.09 | 3.02 |
| 65 | 60.50 | 577 | 268 | 151 | 9.54 | 4.43 | 2.50 |
| 66 | 120.50 | 1203 | 622 | 390 | 9.98 | 5.16 | 3.24 |
| 67 | 199.00 | 915 | 521 | 274 | 4.60 | 2.62 | 1.38 |
| 68 | 50.00 | 595 | 183 | 118 | 11.90 | 3.66 | 2.36 |
| 69 | 127.50 | 1458 | 592 | 366 | 11.44 | 4.64 | 2.87 |
| 70 | 142.50 | 323 | 89 | 46 | 2.27 | 0.62 | 0.32 |
| 71 | 123.50 | 1261 | 460 | 324 | 10.21 | 3.72 | 2.62 |
| 72 | 65.00 | 195 | 80 | 55 | 3.00 | 1.23 | 0.85 |
| 73 | 92.00 | 1035 | 413 | 238 | 11.25 | 4.49 | 2.59 |
| 74 | 128.00 | 1500 | 638 | 307 | 11.72 | 4.98 | 2.40 |
| 75 | 31.50 | 289 | 153 | 81 | 9.17 | 4.86 | 2.57 |
| 76 | 149.00 | 1302 | 631 | 331 | 8.74 | 4.23 | 2.22 |
| 77 | 57.00 | 605 | 271 | 166 | 10.61 | 4.75 | 2.91 |
| 78 | 94.50 | 1097 | 448 | 247 | 11.61 | 4.74 | 2.61 |
| 79 | 58.50 | 704 | 259 | 126 | 12.03 | 4.43 | 2.15 |
| 80 | 131.00 | 709 | 625 | 236 | 5.41 | 4.77 | 1.80 |
| 81 | 42.50 | 489 | 183 | 126 | 11.51 | 4.31 | 2.96 |
| 82 | 25.50 | 284 | 124 | 89 | 11.14 | 4.86 | 3.49 |
| 83 | 32.50 | 383 | 166 | 110 | 11.78 | 5.11 | 3.38 |
| 84 | 48.00 | 488 | 173 | 117 | 10.17 | 3.60 | 2.44 |
| 85 | 48.00 | 376 | 194 | 105 | 7.83 | 4.04 | 2.19 |
| 86 | 56.00 | 499 | 300 | 154 | 8.91 | 5.36 | 2.75 |
| 87 | 49.00 | 583 | 214 | 106 | 11.90 | 4.37 | 2.16 |
| 88 | 35.50 | 445 | 143 | 73 | 12.54 | 4.03 | 2.06 |
| 89 | 44.00 | 454 | 207 | 121 | 10.32 | 4.70 | 2.75 |
| 90 | 24.00 | 258 | 115 | 59 | 10.75 | 4.79 | 2.46 |
| 91 | 30.00 | 369 | 149 | 83 | 12.30 | 4.97 | 2.77 |
| 92 | 49.00 | 592 | 176 | 93 | 12.08 | 3.59 | 1.90 |
| 93 | 7.00 | 82 | 30 | 26 | 11.71 | 4.29 | 3.71 |
| 94 | 101.50 | 1235 | 503 | 324 | 12.17 | 4.96 | 3.19 |
| 95 | 26.00 | 322 | 134 | 76 | 12.38 | 5.15 | 2.92 |
| 96 | 21.00 | 239 | 87 | 71 | 11.38 | 4.14 | 3.38 |
| 97 | 40.50 | 481 | 177 | 108 | 11.88 | 4.37 | 2.67 |
| 98 | 90.50 | 886 | 410 | 210 | 9.79 | 4.53 | 2.32 |
| 99 | 29.00 | 243 | 138 | 81 | 8.38 | 4.76 | 2.79 |
| 100 | 26.00 | 309 | 96 | 48 | 11.88 | 3.69 | 1.85 |
| 101 | 79.00 | 609 | 310 | 173 | 7.71 | 3.92 | 2.19 |
| 102 | 21.50 | 234 | 117 | 58 | 10.88 | 5.44 | 2.70 |
| 103 | 71.00 | 871 | 309 | 165 | 12.27 | 4.35 | 2.32 |
| 104 | 41.50 | 428 | 202 | 130 | 10.31 | 4.87 | 3.13 |
| 105 | 30.50 | 390 | 131 | 81 | 12.79 | 4.30 | 2.66 |
| 106 | 52.50 | 590 | 278 | 160 | 11.24 | 5.30 | 3.05 |
| 107 | 8.50 | 72 | 31 | 19 | 8.47 | 3.65 | 2.24 |
| **Mean** | 72.74 | 668.10 | 298.90 | 172.80 | 9.64 | 4.19 | 2.46 |
| **SE** | 4.68 | 42.64 | 20.40 | 12.18 | 0.25 | 0.10 | 0.06 |

**Table S3 | Statistics of sleep duration, SO, spindle and coupling event numbers and densities for all 107 subjects during N2&N3 stage.**
