# Supplementary material for: Human brain-wide activation of sleep rhythms
Source: eLife. 2026 Aug 12;14:RP103956. doi: 10.7554/eLife.103956 (PMC13466540; doi:10.7554/eLife.103956)
Supplement: Supplementary file 5 [file elife-103956-supp4.docx]

| **Subject ID** | **REM Sleep Stage** | | | | | | |
| --- | --- | --- | --- | --- | --- | --- | --- |
|  | **Time (min)** | **Number of SO** | **Number of spindle** | **Number of coupling** | **Density of SO (times/min)** | **Density of spindle (times/min)** | **Density of coupling (times/min)** |
| 1 | 2.00 | 3 | 7 | 1 | 1.50 | 3.50 | 0.50 |
| 2 | 4.00 | 15 | 13 | 5 | 3.75 | 3.25 | 1.25 |
| 3 | 4.50 | 14 | 8 | 4 | 3.11 | 1.78 | 0.89 |
| 4 | 7.00 | 0 | 0 | 0 | 0.00 | 0.00 | 0.00 |
| 5 | 3.50 | 11 | 11 | 2 | 3.14 | 3.14 | 0.57 |
| 6 | 24.50 | 28 | 29 | 3 | 1.14 | 1.18 | 0.12 |
| 7 | 5.00 | 0 | 0 | 0 | 0.00 | 0.00 | 0.00 |
| 8 | 21.00 | 5 | 15 | 2 | 0.24 | 0.71 | 0.10 |
| 9 | 4.50 | 0 | 0 | 0 | 0.00 | 0.00 | 0.00 |
| 10 | 15.00 | 39 | 19 | 6 | 2.60 | 1.27 | 0.40 |
| 11 | 4.50 | 0 | 0 | 0 | 0.00 | 0.00 | 0.00 |
| 12 | 4.50 | 12 | 6 | 1 | 2.67 | 1.33 | 0.22 |
| 13 | 2.00 | 0 | 0 | 0 | 0.00 | 0.00 | 0.00 |
| 14 | 0.50 | 0 | 0 | 0 | 0.00 | 0.00 | 0.00 |
| 15 | 16.00 | 38 | 36 | 4 | 2.38 | 2.25 | 0.25 |
| 16 | 11.50 | 8 | 20 | 3 | 0.70 | 1.74 | 0.26 |
| 17 | 12.50 | 5 | 18 | 1 | 0.40 | 1.44 | 0.08 |
| 18 | 4.00 | 0 | 0 | 0 | 0.00 | 0.00 | 0.00 |
| 19 | 11.50 | 59 | 20 | 10 | 5.13 | 1.74 | 0.87 |
| 20 | 5.50 | 31 | 15 | 5 | 5.64 | 2.73 | 0.91 |
| 21 | 12.00 | 19 | 34 | 5 | 1.58 | 2.83 | 0.42 |
| 22 | 0.50 | 0 | 0 | 0 | 0.00 | 0.00 | 0.00 |
| 23 | 11.50 | 0 | 0 | 0 | 0.00 | 0.00 | 0.00 |
| 24 | 8.00 | 28 | 4 | 4 | 3.50 | 0.50 | 0.50 |
| 25 | 32.50 | 11 | 3 | 1 | 0.34 | 0.09 | 0.03 |
| 26 | 2.50 | 6 | 6 | 1 | 2.40 | 2.40 | 0.40 |
| 27 | 7.50 | 40 | 8 | 3 | 5.33 | 1.07 | 0.40 |
| 28 | 5.50 | 12 | 17 | 7 | 2.18 | 3.09 | 1.27 |
| 29 | 17.50 | 23 | 69 | 13 | 1.31 | 3.94 | 0.74 |
| 30 | 6.00 | 26 | 16 | 6 | 4.33 | 2.67 | 1.00 |
| 31 | 13.00 | 30 | 12 | 4 | 2.31 | 0.92 | 0.31 |
| 32 | 0.50 | 0 | 0 | 0 | 0.00 | 0.00 | 0.00 |
| 33 | 1.00 | 0 | 0 | 0 | 0.00 | 0.00 | 0.00 |
| 34 | 9.50 | 42 | 7 | 1 | 4.42 | 0.74 | 0.11 |
| 35 | 2.50 | 10 | 9 | 4 | 4.00 | 3.60 | 1.60 |
| 36 | 9.50 | 37 | 21 | 6 | 3.89 | 2.21 | 0.63 |
| 37 | 24.00 | 76 | 40 | 9 | 3.17 | 1.67 | 0.38 |
| 38 | 4.00 | 2 | 6 | 2 | 0.50 | 1.50 | 0.50 |
| 39 | 13.00 | 49 | 19 | 5 | 3.77 | 1.46 | 0.38 |
| 40 | 8.50 | 15 | 18 | 4 | 1.76 | 2.12 | 0.47 |
| 41 | 7.00 | 8 | 16 | 4 | 1.14 | 2.29 | 0.57 |
| 42 | 17.00 | 45 | 67 | 11 | 2.65 | 3.94 | 0.65 |
| 43 | 25.00 | 27 | 107 | 14 | 1.08 | 4.28 | 0.56 |
| 44 | 15.50 | 33 | 55 | 5 | 2.13 | 3.55 | 0.32 |
| 45 | 7.50 | 18 | 21 | 5 | 2.40 | 2.80 | 0.67 |
| 46 | 0.50 | 0 | 0 | 0 | 0.00 | 0.00 | 0.00 |
| 47 | 1.00 | 0 | 0 | 0 | 0.00 | 0.00 | 0.00 |
| 48 | 46.00 | 129 | 236 | 49 | 2.80 | 5.13 | 1.07 |
| 49 | 0.50 | 0 | 0 | 0 | 0.00 | 0.00 | 0.00 |
| 50 | 29.50 | 69 | 74 | 19 | 2.34 | 2.51 | 0.64 |
| 51 | 19.50 | 49 | 43 | 11 | 2.51 | 2.21 | 0.56 |
| 52 | 8.00 | 0 | 0 | 0 | 0.00 | 0.00 | 0.00 |
| 53 | 6.00 | 20 | 5 | 2 | 3.33 | 0.83 | 0.33 |
| 54 | 6.00 | 5 | 24 | 4 | 0.83 | 4.00 | 0.67 |
| 55 | 1.00 | 0 | 0 | 0 | 0.00 | 0.00 | 0.00 |
| 56 | 20.00 | 32 | 21 | 5 | 1.60 | 1.05 | 0.25 |
| 57 | 0.50 | 0 | 0 | 0 | 0.00 | 0.00 | 0.00 |
| 58 | 6.00 | 14 | 22 | 3 | 2.33 | 3.67 | 0.50 |
| 59 | 45.00 | 220 | 146 | 65 | 4.89 | 3.24 | 1.44 |
| 60 | 0.50 | 0 | 0 | 0 | 0.00 | 0.00 | 0.00 |
| 61 | 8.00 | 0 | 0 | 0 | 0.00 | 0.00 | 0.00 |
| 62 | 10.50 | 41 | 12 | 5 | 3.90 | 1.14 | 0.48 |
| 63 | 5.50 | 19 | 13 | 2 | 3.45 | 2.36 | 0.36 |
| 64 | 7.50 | 20 | 41 | 6 | 2.67 | 5.47 | 0.80 |
| 65 | 9.00 | 15 | 35 | 2 | 1.67 | 3.89 | 0.22 |
| 66 | 22.00 | 58 | 32 | 13 | 2.64 | 1.45 | 0.59 |
| 67 | 22.00 | 108 | 63 | 19 | 4.91 | 2.86 | 0.86 |
| 68 | 18.50 | 101 | 16 | 5 | 5.46 | 0.86 | 0.27 |
| 69 | 5.50 | 13 | 10 | 1 | 2.36 | 1.82 | 0.18 |
| 70 | 10.00 | 13 | 15 | 2 | 1.30 | 1.50 | 0.20 |
| 71 | 29.50 | 69 | 69 | 15 | 2.34 | 2.34 | 0.51 |
| 72 | 0.00 | 0 | 0 | 0 | 0.00 | 0.00 | 0.00 |
| 73 | 14.50 | 44 | 6 | 4 | 3.03 | 0.41 | 0.28 |
| 74 | 13.50 | 45 | 27 | 12 | 3.33 | 2.00 | 0.89 |
| 75 | 3.50 | 8 | 5 | 1 | 2.29 | 1.43 | 0.29 |
| 76 | 4.50 | 6 | 2 | 1 | 1.33 | 0.44 | 0.22 |
| 77 | 14.00 | 49 | 23 | 11 | 3.50 | 1.64 | 0.79 |
| 78 | 26.00 | 86 | 25 | 5 | 3.31 | 0.96 | 0.19 |
| 79 | 2.50 | 8 | 9 | 2 | 3.20 | 3.60 | 0.80 |
| 80 | 6.50 | 4 | 10 | 1 | 0.62 | 1.54 | 0.15 |
| 81 | 4.00 | 2 | 15 | 1 | 0.50 | 3.75 | 0.25 |
| 82 | 3.00 | 13 | 10 | 4 | 4.33 | 3.33 | 1.33 |
| 83 | 10.50 | 34 | 26 | 8 | 3.24 | 2.48 | 0.76 |
| 84 | 1.00 | 6 | 1 | 1 | 6.00 | 1.00 | 1.00 |
| 85 | 8.00 | 22 | 28 | 6 | 2.75 | 3.50 | 0.75 |
| 86 | 0.00 | 0 | 0 | 0 | 0.00 | 0.00 | 0.00 |
| 87 | 3.50 | 4 | 5 | 1 | 1.14 | 1.43 | 0.29 |
| 88 | 1.50 | 9 | 2 | 1 | 6.00 | 1.33 | 0.67 |
| 89 | 8.50 | 13 | 29 | 3 | 1.53 | 3.41 | 0.35 |
| 90 | 4.00 | 22 | 11 | 3 | 5.50 | 2.75 | 0.75 |
| 91 | 1.50 | 8 | 4 | 2 | 5.33 | 2.67 | 1.33 |
| 92 | 2.00 | 5 | 8 | 1 | 2.50 | 4.00 | 0.50 |
| 93 | 1.50 | 0 | 0 | 0 | 0.00 | 0.00 | 0.00 |
| 94 | 5.50 | 10 | 22 | 3 | 1.82 | 4.00 | 0.55 |
| 95 | 6.00 | 6 | 17 | 2 | 1.00 | 2.83 | 0.33 |
| 96 | 7.00 | 20 | 23 | 4 | 2.86 | 3.29 | 0.57 |
| 97 | 0.00 | 0 | 0 | 0 | 0.00 | 0.00 | 0.00 |
| 98 | 11.00 | 12 | 26 | 3 | 1.09 | 2.36 | 0.27 |
| 99 | 15.00 | 20 | 24 | 2 | 1.33 | 1.60 | 0.13 |
| 100 | 4.50 | 5 | 18 | 2 | 1.11 | 4.00 | 0.44 |
| 101 | 3.50 | 4 | 8 | 2 | 1.14 | 2.29 | 0.57 |
| 102 | 7.50 | 27 | 19 | 7 | 3.60 | 2.53 | 0.93 |
| 103 | 1.00 | 0 | 0 | 0 | 0.00 | 0.00 | 0.00 |
| 104 | 8.50 | 16 | 33 | 8 | 1.88 | 3.88 | 0.94 |
| 105 | 6.50 | 0 | 0 | 0 | 0.00 | 0.00 | 0.00 |
| 106 | 2.50 | 8 | 11 | 3 | 3.20 | 4.40 | 1.20 |
| 107 | 16.50 | 80 | 6 | 1 | 4.85 | 0.36 | 0.06 |
| **Mean** | 9.27 | 22.58 | 19.64 | 4.62 | 2.07 | 1.81 | 0.43 |
| **SE** | 0.87 | 3.08 | 2.99 | 0.81 | 0.17 | 0.14 | 0.04 |

**Table S4 | Statistics of sleep duration, SO, spindle and coupling event numbers and densities for all 107 subjects during REM stage.**
