# Supplementary material for: Human brain-wide activation of sleep rhythms
Source: eLife. 2026 Aug 12;14:RP103956. doi: 10.7554/eLife.103956 (PMC13466540; doi:10.7554/eLife.103956)
Supplement: Supplementary file 6 [file elife-103956-supp5.docx]

| **Brain areas** | **Laterality** | **Peak T-value** | **Cluster size** | **Peak coordinate (MNI)** | | |
| --- | --- | --- | --- | --- | --- | --- |
|  |  |  |  | **x** | **y** | **z** |
| Anterior Supramarginal Gyrus | L | -3.93 | 303 | -52 | -29 | 40 |
| Anterior Supramarginal Gyrus | R | -2.66 | 18 | 67 | -29 | 27 |
| Superior Frontal Gyrus | L | -4.53 | 2766 | -12 | 4 | 66 |
| Superior Frontal Gyrus | R | -4.86 | 2122 | 12 | 21 | 59 |
| Middle Frontal Gyrus | L | -5.57 | 3320 | -44 | 26 | 33 |
| Middle Frontal Gyrus | R | -5.56 | 3312 | 40 | 25 | 37 |
| Anterior Paracingulate Gyrus | L | -7.28 | 4153 | -5 | 38 | -10 |
| Anterior Paracingulate Gyrus | R | -7.13 | 3918 | 4 | 45 | -8 |
| Angular Gyrus | L | -3.52 | 519 | -62 | -54 | 26 |
| Angular Gyrus | R | -2.66 | 401 | 50 | -52 | 49 |
| Frontal Orbital Cortex | L | -6.46 | 6392 | -38 | 33 | -17 |
| Frontal Orbital Cortex | R | -7.26 | 5637 | 28 | 28 | -20 |
| Superior Parietal Lobule | L | -5.10 | 1774 | -35 | -44 | 58 |
| Superior Parietal Lobule | R | -5.96 | 1457 | 35 | -41 | 59 |
| Insular Cortex | L | -4.89 | 1004 | -32 | -26 | 16 |
| Insular Cortex | R | -6.34 | 1028 | 34 | -24 | 9 |
| Posterior Cingulate Gyrus | L | -10.83 | 4525 | -5 | -47 | 19 |
| Posterior Cingulate Gyrus | R | -10.47 | 5838 | 5 | -43 | 27 |
| Posterior Supramarginal Gyrus | L | -3.24 | 102 | -61 | -51 | 26 |
| Posterior Supramarginal Gyrus | R | -2.41 | 7 | 67 | -38 | 21 |

**Table S5 | Peak and significant cluster of fMRI activity during SO main effect.** We used the SO main effect whole-brain activation patterns in **Figure 3b.** ROIs were defined anatomically (see Methods). Cluster sizes are reported *p_unc._* < 0.001.
