# Supplementary material for: Human brain-wide activation of sleep rhythms
Source: eLife. 2026 Aug 12;14:RP103956. doi: 10.7554/eLife.103956 (PMC13466540; doi:10.7554/eLife.103956)
Supplement: Supplementary file 7 [file elife-103956-supp6.docx]

| **Brain areas** | **Laterality** | **Peak T-value** | **Cluster size** | **Peak coordinate (MNI)** | | |
| --- | --- | --- | --- | --- | --- | --- |
|  |  |  |  | **x** | **y** | **z** |
| Anterior Supramarginal Gyrus | L | -6.36 | 1357 | -55 | -29 | 45 |
| Anterior Supramarginal Gyrus | R | -6.53 | 1671 | 55 | -26 | 49 |
| Superior Frontal Gyrus | L | -6.83 | 2274 | -20 | 33 | 38 |
| Superior Frontal Gyrus | R | 5.52 | 2320 | 12 | -4 | 75 |
| Middle Frontal Gyrus | L | -8.03 | 5350 | -42 | 30 | 30 |
| Middle Frontal Gyrus | R | -5.77 | 4194 | 31 | 28 | 41 |
| Anterior Paracingulate Gyrus | L | 7.15 | 1086 | -7 | 11 | 42 |
| Anterior Paracingulate Gyrus | R | 6.68 | 2222 | 2 | 15 | 41 |
| Angular Gyrus | L | -7.55 | 1240 | -48 | -58 | 50 |
| Angular Gyrus | R | -7.33 | 4588 | 53 | -53 | 34 |
| Frontal Orbital Cortex | L | -4.61 | 1149 | -41 | 21 | -11 |
| Frontal Orbital Cortex | R | -3.98 | 645 | 40 | 20 | -16 |
| Superior Parietal Lobule | L | -6.91 | 1687 | -36 | -56 | 61 |
| Superior Parietal Lobule | R | -7.79 | 3091 | 42 | -43 | 62 |
| Insular Cortex | L | -6.99 | 1103 | -37 | -20 | 15 |
| Insular Cortex | R | -7.26 | 2169 | 36 | -22 | 12 |
| Posterior Cingulate Gyrus | L | -7.85 | 3208 | -5 | -46 | 38 |
| Posterior Cingulate Gyrus | R | -7.10 | 3352 | 0 | -45 | 36 |
| Posterior Supramarginal Gyrus | L | -6.71 | 1263 | -54 | -49 | 39 |
| Posterior Supramarginal Gyrus | R | -6.61 | 2376 | 48 | -38 | 54 |

**Table S6 | Peak and significant cluster of fMRI activity during spindle main effect.** We used the Spindle main effect whole-brain activation patterns in **Figure 3c.** ROIs were defined anatomically (see Methods). Cluster sizes are reported *p_unc._* < 0.001.
