# Supplementary material for: Human brain-wide activation of sleep rhythms
Source: eLife. 2026 Aug 12;14:RP103956. doi: 10.7554/eLife.103956 (PMC13466540; doi:10.7554/eLife.103956)
Supplement: Supplementary file 8 [file elife-103956-supp7.docx]

| **Brain areas** | **Laterality** | **Peak T-value** | **Cluster size** | **Peak coordinate (MNI)** | | |
| --- | --- | --- | --- | --- | --- | --- |
|  |  |  |  | **x** | **y** | **z** |
| Anterior Supramarginal Gyrus | L | -4.95 | 1327 | -56 | -33 | 38 |
| Anterior Supramarginal Gyrus | R | -2.42 | 10 | 60 | -23 | 27 |
| Superior Frontal Gyrus | L | -3.50 | 398 | -14 | 10 | 64 |
| Superior Frontal Gyrus | R | -4.44 | 596 | 6 | 23 | 49 |
| Middle Frontal Gyrus | L | -4.74 | 2455 | -50 | 11 | 40 |
| Middle Frontal Gyrus | R | -6.01 | 1694 | 53 | 17 | 37 |
| Anterior Paracingulate Gyrus | L | -3.56 | 381 | -1 | 21 | 47 |
| Anterior Paracingulate Gyrus | R | -4.98 | 725 | 7 | 21 | 47 |
| Angular Gyrus | L | 1.71 | 0 | -62 | -54 | 26 |
| Angular Gyrus | R | 2.63 | 149 | 55 | -58 | 19 |
| Frontal Orbital Cortex | L | -4.72 | 1492 | -47 | 26 | -6 |
| Frontal Orbital Cortex | R | -4.24 | 872 | 40 | 27 | -2 |
| Superior Parietal Lobule | L | -4.83 | 1177 | -37 | -46 | 56 |
| Superior Parietal Lobule | R | -4.02 | 584 | 28 | -50 | 54 |
| Insular Cortex | L | -4.01 | 1385 | -38 | 19 | -1 |
| Insular Cortex | R | -4.36 | 1374 | 32 | 21 | 0 |
| Posterior Cingulate Gyrus | L | -2.93 | 83 | -9 | -32 | 40 |
| Posterior Cingulate Gyrus | R | -2.91 | 130 | 7 | -30 | 42 |
| Posterior Supramarginal Gyrus | L | -1.96 | 0 | -48 | -44 | 46 |
| Posterior Supramarginal Gyrus | R | -2.02 | 0 | 49 | -37 | 51 |

**Table S7 | Peak and significant cluster of fMRI activity during SO-spindle interaction.** We used the SO-spindle interaction effect whole-brain activation patterns in **Figure 3d.** ROIs were defined anatomically (see Methods). Cluster sizes are reported *p_unc._* < 0.001.
