# Supplementary material for: Human brain-wide activation of sleep rhythms
Source: eLife. 2026 Aug 12;14:RP103956. doi: 10.7554/eLife.103956 (PMC13466540; doi:10.7554/eLife.103956)
Supplement: Peer review history

# Editor report: eLife Assessment

**DOI:** 10.7554/eLife.103956.4.sa0

**Authors:** Simon van Gaal

**Affiliations:** University of Amsterdam Netherlands

**Keywords:** Important, Solid

The study presents **important** findings from a very rich EEG-fMRI dataset including 107 participants, which was collected during nocturnal naps. Using overall **solid** methods, the authors link activity in memory related brain regions (e.g., hippocampus, thalamus, and medial prefrontal cortex), as well as their functional connectivity, to the occurrence of canonical sleep rhythms (spindles and slow oscillations) during non-rapid eye movement (NREM) sleep. This work will be of broad interest to researchers studying sleep, memory, and related domains.

# Referee report: Reviewer #1 (Public review)

**DOI:** 10.7554/eLife.103956.4.sa1

Wang et al., recorded concurrent EEG-fMRI in 107 participants during nocturnal NREM sleep to investigate brain activity and connectivity related to slow oscillations (SO), sleep spindles, and in particular their co-occurrence. The authors found SO-spindle coupling to be correlated with increased thalamic and hippocampal activity, and with increased functional connectivity from the hippocampus to the thalamus and from the thalamus to the neocortex, especially the medial prefrontal cortex (mPFC). They concluded the brain-wide activation pattern to resemble episodic memory processing, but to be dissociated from task-related processing and suggest that the thalamus plays a crucial role in coordinating the hippocampal-cortical dialogue during sleep.

The paper offers an impressively large and highly valuable dataset that provides the opportunity for gaining important new insights into the network substrate involved in SOs, spindles, and their coupling.

Comments on latest version:

The authors have substantially revised their manuscript and sufficiently addressed all of my previous concerns. I have no further comments.

# Author comment: Author response

**DOI:** 10.7554/eLife.103956.4.sa2

**Authors:** Haiteng Wang, Qihong Zou, Jinbo Zhang, Jia-Hong Gao, Yunzhe Liu

**Affiliations:** Beijing Normal University Beijing China; Center for MRI Research, Academy for Advanced Interdisciplinary Studies, Peking University Beijing China; Beijing Normal University Beijing China; Peking University Beijing China; Beijing Normal University Beijing China

The following is the authors’ response to the previous reviews

**Public Reviews:**

**Reviewer #1 (Public review):**

Wang et al., recorded concurrent EEG-fMRI in 107 participants during nocturnal NREM sleep to investigate brain activity and connectivity related to slow oscillations (SO), sleep spindles, and in particular their co-occurrence. The authors found SO-spindle coupling to be correlated with increased thalamic and hippocampal activity, and with increased functional connectivity from the hippocampus to the thalamus and from the thalamus to the neocortex, especially the medial prefrontal cortex (mPFC). They concluded the brain-wide activation pattern to resemble episodic memory processing, but to be dissociated from task-related processing and suggest that the thalamus plays a crucial role in coordinating the hippocampal-cortical dialogue during sleep.

The paper offers an impressively large and highly valuable dataset that provides the opportunity for gaining important new insights into the network substrate involved in SOs, spindles, and their coupling.

Thank you for this encouraging assessment. We appreciate your recognition of the value of the dataset and of the questions it allows us to address. Below, we respond to each of your points directly and revise the manuscript accordingly.

Comments on revisions:

Re 1: The revised introduction now cites a couple of papers but discusses them only very superficially, lumping together several studies with very different key results. This is still not very informative for the reader and does not sufficiently acknowledge previously published work. Here are two examples to illustrate this:

(a) "These studies have generally reported that slow oscillations are associated with widespread cortical and subcortical BOLD changes, whereas spindles elicit activation in the thalamus, as well as in several cortical and paralimbic regions." Several studies even showed e.g., a clear activation of the hippocampus and parahippocampal gyrus associated with spindles, not just the thalamus

Thank you for this comment. We agree that our previous sentence was too broad and did not sufficiently reflect the range of findings in the sleep literature. We have therefore rewritten the Introduction to state explicitly that spindle-related BOLD changes have been reported not only in the thalamus, but also in cortical and paralimbic regions, including the hippocampus and parahippocampal gyrus (Bergmann et al., 2012; Caporro et al., 2012; Fogel et al., 2017; Schabus et al., 2007).

Introduction, Page 3-4, Lines 58-62

“Consistent with this view, prior human EEG-fMRI studies have reported spindle-related activation not only in the thalamus, but also in the hippocampus and adjacent parahippocampal gyrus (Bergmann et al., 2012; Schabus et al., 2007). Spindle-related activity has also been linked to striatal engagement, suggesting a broader network that may support memory-related processing during sleep (Fogel et al., 2017).”

Introduction, Page 4, Lines 71-78

“Previous EEG-fMRI studies on sleep have examined both global sleep characteristics (Hale et al., 2016; Moehlman et al., 2019) and the neural correlates of specific waves, including slow oscillations and spindles. These studies have generally shown that slow oscillations are associated with widespread cortical and subcortical BOLD changes (Czisch et al., 2009; Ilhan-Bayrakcı et al., 2022; Picchioni et al., 2011), whereas spindles have been linked not only to thalamic activation but also to cortical and paralimbic regions, including the hippocampus and parahippocampal gyrus (Bergmann et al., 2012; Caporro et al., 2012; Fogel et al., 2017; Schabus et al., 2007).”

Introduction, Page 5, Lines 103-106

“This coupling was associated with increased activation in both the thalamus and hippocampus, with functional connectivity patterns suggesting thalamic coordination of hippocampal-cortical communication, in line with prior EEG-fMRI studies of spindle-related activity (Bergmann et al., 2012; Caporro et al., 2012; Fogel et al., 2017; Schabus et al., 2007).”

(b) "Although these findings provide valuable insights into the BOLD correlates of sleep rhythms, they often do not employ sophisticated temporal modeling (Huang et al., 2024) [, ...]." - previous studies have used e.g., spindle event-related regressors with individual spindle amplitudes as parametric modulators, first and second order derivatives of the HRF function, as well as PPI connectivity analyses, which I would consider rather sophisticated temporal modelling.

We agree that several previous studies have already employed sophisticated modelling approaches, including parametric modulation, HRF derivatives, and PPI analyses (Bergmann et al., 2012; Caporro et al., 2012; Fogel et al., 2017; Picchioni et al., 2011). Our intention was not to suggest that such methods are absent from the literature.

Rather, we aimed to highlight that most prior work has focused on modelling individual SO or spindle events, whereas explicit modelling of their temporal interaction (e.g., SO-spindle coupling) has been less commonly addressed. We have revised the sentence to clarify this point more precisely.

Introduction, Page 4 Lines 78-82

“Although these findings provide important insight into the BOLD correlates of sleep rhythms, most previous studies have focused on individual oscillatory events rather than explicitly modelling their temporal interaction (Bergmann et al., 2012; Caporro et al., 2012; Fogel et al., 2017; Picchioni et al., 2011). Only a few recent studies have begun to examine coupling between rhythms directly, for example Huang et al. (2024).”

Re 4+9: The short overall recordings in some subjects on the one hand and the large number of spindles and SOs detected in N1 sleep stages are still highly concerning, in fact even more so, now that the actual numbers have been provided in the Supplementary Tables. Either the sleep staging or the detection of SO and spindle events must be incorrect. I understand that for specific EEG analysis and fMRI modelling purposes sometimes slightly different thresholds are used as compared to clinical sleep staging, but several parameters here are alarmingly off.

(a) Given that proper NREM sleep (N2+N3) is the relevant stage for the analyses conducted in this paper, some of the N2+N3 durations are very short (eg 7-8 min) while those subjects' results have the same impact on the group level analyses as those with >100 min of N2+N3. Either subjects with very little relevant data (not overall recording time but N2+N3 time) should be excluded or weighting subject data for the group analyses according to the amount od contributed data should be done.

Thank you for the suggestion. It is true that participants with very little N2/3 sleep could contribute noisier subject-level estimates to the group analysis. We therefore checked this directly. Only three participants contributed less than 10 min of N2/3 sleep, and excluding them did not change the main results. For example, hippocampal activation during SO-spindle coupling remained significant after exclusion, *t*_(103)_ = 2.50, *p* = 0.0071, compared with *t*_(106)_ = 2.50, *p* = 0.0070 in the full sample. We have added this control analysis to the Results so that the robustness of the group findings is explicit in the manuscript.

Results, Page 11-12, Lines 238-250

To ensure the results were not driven by individual differences or parameter selection, we conducted a series of control analyses. First, we excluded participants with less than 10 minutes of N2/3 sleep. Only three participants met this criterion, and their exclusion did not change the main results. For example, hippocampal activation during SO-spindle coupling remained significant (*t*_(103)_ = 2.50, *p* = 0.0071), comparable to the full sample (*t*_(106)_ = 2.50, *p* = 0.0070). Second, because the absolute number of detected SO-spindle coupling events depends on the SO detection threshold, we examined whether the main EEG-fMRI results were sensitive to this parameter. To this end, we varied the SO percentile threshold and reconstructed the EEG-informed GLM at each level. Hippocampal activation during SO-spindle coupling remained significant across a range of thresholds (71st-80th percentile; Fig. S6). Third, to test whether the results depended on the use of a single lateralised frontal electrode, we repeated the EEG-informed fMRI GLM using events detected from Fz. Hippocampal activation during SO-spindle coupling again remained significant (*t*_(106)_ = 2.47, *p* = 0.0076), closely matching the original F3-based result (*t*_(106)_ = 2.50, *p* = 0.0070).

(b) The authors argue that the SO and spindle detection algorithms are valid since widely used and that they were developed for N2+N3 stages, which is why they will also detect events in other stages: "While, because the detection methods for SO and spindle are based on percentiles, this method will always detect a certain number of events when used for other stages (N1 and REM) sleep data, but the differences between these events and those detected in stage N23 remain unclear." I do agree that with very liberal thresholds, also SO and spindle vents may be detected in other stages, but it shouldn't be that many. If the percentiles of amplitude thresholds were defined based on properly scored N2+N3 stages only, very few events should be detected (erroneously!) in N1, as the occurrence of K-complexes (isolated SOs) and spindles per definition makes it N2, and during REM sleep only very few spindles and SOs are allowed to occur, without scoring it NREM instead. For the first subject (just as example, but with similar numbers for the rest of the sample), reveals as many as 60 SOs and 31 spindles within 8 min of N1 sleep (Table S2) as well as 13 SOs and 7 spindles within 2 min of REM sleep (Table S4). These numbers are completely unrealistic and question the correctness of the sleep staging as well as the physiological relevance of the EEG graphoelements identified as SO and spindles. It also completely undermines the interpretability of the respective event regressors for the fMRI analyses.

(c) Likely, given the large numbers of coupled SO-spindle events and the apparently very low amplitude criteria for event identification, also the number of SO-spindle couplings is likely severely overestimated.

We thank the reviewer for raising this important point. We agree with you that the original stage-wise percentile thresholding could inflate the apparent number of SOs and spindles outside N2/3 sleep. In the original analysis, the thresholds were estimated separately within each sleep stage. As you point out, this procedure can force the detector to label a relatively large number of events in N1 and REM, even when those waveforms should not be interpreted as canonical N2/3 SOs or spindles. We have therefore revised the detection procedure. Following your concern and Reviewer 2’s suggestion, the SO and spindle thresholds are now defined only from N2/3 sleep within each participant, where SOs and spindles are most abundant and physiologically expected to occur. These fixed N2/3-derived thresholds were then applied unchanged to N1 and REM for descriptive reporting. This avoids the artificial normalisation of event detection across sleep stages that can arise when each stage has its own percentile threshold. And we have revised all relevant sections of the manuscript, including “[Results, Page 6-7 Lines 134-148]; [Fig. 1e]; [Results, Page 9 Lines 175-191]; [Fig. 2b]; [Methods, Page 25-27, Lines 567-604]; [Fig. S2-S4]; [Table S2, S4].”

With this revised procedure, detections outside N2/3 are clearly lower than those in N2/3. The mean densities are 2.95 SOs/min, 2.71 spindles/min, and 0.75 coupling events/min in N1, and 2.07 SOs/min, 1.81 spindles/min, and 0.43 coupling events/min in REM. We agree with you that the remaining detections in N1 and REM should not be treated as physiological equivalents of canonical N2/3 SOs, spindles, or SO-spindle complexes. We therefore report them only as descriptive detector outputs obtained under a fixed N2/3-derived threshold (see Table S2, S4 in the revised manuscript). We do not use them to support any physiological claim about SO-spindle coupling in N1 or REM.

This point is also important for the fMRI analyses. You are right that inflated N1 or REM detections would undermine the interpretability of event regressors if those detections entered the EEG-informed fMRI models. They did not. All EEG-informed fMRI GLM and PPI analyses were restricted to N2/3 sleep, where SOs, spindles, and their coupling are physiologically expected and where the detection thresholds were defined. Thus, the central fMRI event regressors were based only on N2/3 events, not on detections from N1 or REM.

We also agree with you that the absolute number of detected SO-spindle couplings depends on the chosen detection threshold. For this reason, we tested whether the main EEG-fMRI result depended on the specific detector setting. Hippocampal activation during SO-spindle coupling remained significant when the SO detection threshold was varied between the 71st and 80th percentiles, as shown in Fig. S6. We therefore do not argue that the detector provides a uniquely correct absolute count of SOs, spindles, or coupling events in every sleep stage. Our conclusion is more specific. The main N2/3 EEG-fMRI finding is robust across a reasonable range of SO detection thresholds, detections in N1 and REM are reported only descriptively, and the physiological interpretation of SO-spindle coupling is restricted to N2/3 sleep.

Results, Page 6-7 Lines 134-148

“Each sleep stage is characterised by distinct spectral properties and rhythmic waveforms, serving as physiological markers (Fig. 1c). Because SO and spindle detection relies on amplitude-based percentile thresholds, we avoided estimating separate thresholds within each sleep stage. Instead, for each participant, the SO and spindle thresholds were defined from N2/3 sleep only, where these rhythms are most abundant and physiologically expected, and the same fixed thresholds were then applied to N1 and REM for descriptive comparison.”

“Under this fixed N2/3-derived thresholding, detected SOs and spindles were larger and more frequent in N2/3 than in N1 or REM. SO and spindle amplitudes were significantly higher during N2/3 sleep (SO: 25.59 ± 1.49 μV; spindle: 7.39 ± 0.27 μV) than during N1 (SO: 20.15 ± 2.32 μV; spindle: 5.23 ± 0.27 μV) and REM sleep (SO: 19.84 ± 1.22 μV; spindle: 5.60 ± 0.22 μV; all *p* < 1e-4; Fig. 1e, Fig. S2). The corresponding event densities showed the same pattern, with 9.64 ± 0.25 SOs/min and 4.19 ± 0.10 spindles/min in N2/3, compared with 2.95 ± 0.16 SOs/min and 2.71 ± 0.14 spindles/min in N1, and 2.07 ± 0.17 SOs/min and 1.81 ± 0.14 spindles/min in REM (all *p* < 1e-4). We therefore report detections in N1 and REM only as descriptive outputs of the detector under a fixed N2/3-derived criterion, rather than as physiological equivalents of canonical N2/3 SOs or spindles.”

Fig. 1 legend, Page 8, Line 166-172

“e, Amplitudes (μV) of detected SOs (left) and spindles (right) across sleep stages. SO and spindle detection thresholds were defined from N2/3 sleep within each participant and then applied unchanged to N1 and REM for descriptive comparison. Detections in N1 and REM should therefore be interpreted as detector outputs under this fixed N2/3-derived criterion. The SO amplitudes were measured from the 0.16-1.25 Hz filtered EEG data, and spindle amplitudes were measured from the 12-16 Hz filtered EEG data. Each dot represents an individual participant. Error bars indicate SEM. *** *p* < 0.001.”

Results, Page 9 Lines 175-191

“SO-spindle coupling is considered important for sleep-dependent memory consolidation. In the current study, using the same N2/3-derived detection thresholds described above, we found that SO-spindle coupling occurred most frequently during N2/3 sleep (2.46 ± 0.06 events/min). Coupling density was significantly lower in N1 (0.75 ± 0.05 events/min, *t*_(106)_ = 23.54, *p* < 1e-4) and REM sleep (0.43 ± 0.04 events/min, *t*_(106)_ = 31.24, *p* < 1e-4; Fig. 2b, Table S2-S4), consistent with the expected predominance of SO-spindle coupling in NREM sleep (Ngo et al., 2013; Staresina et al., 2015). As with the individual SO and spindle detections, coupling events detected in N1 and REM were retained only for descriptive stage-wise reporting (see Table S2, S4). They were not used to support physiological claims about SO-spindle coupling in these stages, and they were not entered into the EEG-informed fMRI analyses. All subsequent fMRI GLM and PPI analyses were restricted to N2/3 sleep.”

“After extracting all N2/3 EEG epochs in which SO-spindle coupling occurred, we analysed their spectral and phase characteristics. The spindles were most likely to occur slightly before the UP-state peak of SOs (Fig. 2a, e), aligning with results from both animal studies (Maingret et al., 2016) and human research (Staresina et al., 2015). In our data, this pattern was consistent across subjects (Fig. 2d, Rayleigh test: *z* = 9.51, *p* < 1e-4), with the peak of the spindle aligned at an SO phase of −41.61 ± 0.86° (the SO UP-state peak is 0°).”

Fig. 2 legend, Page 10, Line 202-205

“b, SO-spindle coupling density across sleep stages, using SO and spindle detections obtained with fixed N2/3-derived thresholds. Coupling events in N1 and REM are shown only for descriptive comparison. The EEG-informed fMRI analyses used N2/3 coupling events only.”

Results, Page 11-12, Lines 242-247

“Second, because the absolute number of detected SO-spindle coupling events depends on the SO detection threshold, we examined whether the main EEG-fMRI results were sensitive to this parameter. To this end, we varied the SO percentile threshold and reconstructed the EEG-informed GLM at each level. Hippocampal activation during SO-spindle coupling remained significant across a range of thresholds (71st - 80th percentile; Fig. S6).”

Methods, Page 25-26, Lines 567-575

“Detection of SOs. Data were first bandpass-filtered between 0.16 and 1.25 Hz (Butterworth filter, order 3, bidirectional filtering for zero phase). After identifying all positive-to-negative zero crossings, potential SOs were defined based on the interval between consecutive zero crossings, ranging from 0.8 s to 3 s. For each potential SO, we calculated the amplitude range as the peak minus the trough. For each participant, the amplitude threshold was defined as the 75th percentile of candidate SO amplitude ranges observed during N2/3 sleep. This fixed N2/3-derived threshold was then applied unchanged across the recording for descriptive stage-wise summaries. Detected events were assigned to N1, N2/3 or REM according to the sleep-stage label at the event time. Only candidates exceeding this threshold were labelled as SOs, following previous work (Schreiner et al., 2021).”

Methods, Page 26, Lines 576-583

“Detection of sleep spindles. Detection of sleep spindles. Data were bandpass-filtered between 12 and 16 Hz (Butterworth filter, order 3, bidirectional filtering for zero phase). The root mean square (RMS) of the filtered signal was computed with a 200 ms sliding time window. For each participant, the spindle threshold was defined as the 75th percentile of RMS values observed during N2/3 sleep. This fixed N2/3-derived threshold was then applied unchanged across the recording for descriptive stage-wise summaries. Detected events were assigned to N1, N2/3 or REM according to the sleep-stage label at the event time. RMS segments exceeding this threshold for 0.5 s to 3 s were identified as spindles (Staresina et al., 2015).”

Methods, Page 26, Lines 584-591

“Detection of SO-spindle couplings. From the detected SOs and spindles, we identified the peak time of each spindle. Within each SO interval, we checked whether a spindle peak occurred; if so, that SO was labelled as an SO-spindle coupling event. For descriptive stage-wise summaries, coupling events were assigned to the sleep stage of the corresponding SO trough. For every SO-spindle coupling event, an epoch was created time-locked to the SO trough as the central reference, following Schreiner et al. (2021). We extracted data in a [−4 s to 4 s] window around this point, forming the epoch for each coupling event. For the EEG-informed fMRI analyses, only SO, spindle and SO-spindle coupling events detected during N2/3 sleep were used.”

Methods, Page 26-27, Lines 592-604

“The detection procedures described above were developed primarily for N2 and N3 sleep, where SOs, spindles and their coupling are physiologically expected and most reliably observed (Hahn et al., 2020; Helfrich et al., 2019; Helfrich et al., 2018; Ngo, Fell, & Staresina, 2020; Schreiner et al., 2022; Schreiner et al., 2021; Staresina et al., 2015; Staresina et al., 2023). Because percentile-based thresholds can otherwise force the detector to label events in every sleep stage, we did not estimate separate thresholds within N1 or REM. Instead, for each participant, all SO and spindle thresholds were defined from N2/3 sleep and then applied uniformly across the recording. Tables S1 and S3 report detailed statistical information on sleep rhythm and N2/3 events detection. The N1 and REM events detection reported in Tables S2 and S4, and illustrated in Fig. S2-S4, should therefore be interpreted as descriptive detector outputs under this fixed N2/3-derived criterion, rather than as evidence for canonical N2/3 SOs, spindles or physiological SO-spindle complexes in those stages. These detections were not used in the EEG-informed fMRI GLM or PPI analyses, which were restricted to N2/3 sleep.”

Re 10: The rationale for using a lateralized frontal electrode (F3) for both SO (should have been at least bilateral or central) and spindle detection (should have been a centro-parietal electrode) is not convincing. Other EEG-fMRI spindle or SO papers have used a number of frontal (SO) or centro-parietal (spindles) electrodes averaged or even approaches including all EEG electrodes. Searching events with low thresholds at suboptimal recording sites does not dot this highly valuable dataset justice.

We thank the reviewer for this important comment. We agree that this choice is more sensitive to frontal SOs than to the centro-parietal fast spindle component. Our choice of F3 was driven by the practical constraints of prolonged nocturnal EEG-fMRI recordings. In our MR-compatible EEG setup, FCz was used as the online reference. Central electrodes close to FCz can have reduced signal contrast relative to the reference, and electrodes near the vertex are also more vulnerable to prolonged pressure against the MRI head coil when participants sleep supine for several hours. In this setting, frontal electrodes provided more stable signal quality across the recording. Because the EEG events were used primarily as temporal markers for fMRI modelling, our priority was to obtain reliable event timing during N2/3 sleep rather than to estimate the full scalp topography of SOs and spindles.

We also agree with your concern that this valuable dataset would ideally be analysed with multichannel detection strategies. To test whether the main result depended on the single lateralised F3 site, we repeated the main EEG-informed fMRI analysis using Fz, a midline frontal electrode. The result was unchanged. Hippocampal activation during SO-spindle coupling remained significant when events were detected from Fz, *t*_(106)_ = 2.47, *p* = 0.0076, closely matching the original F3-based result, *t*_(106)_ = 2.50, *p* = 0.0070. This control analysis does not remove the limitation that centro-parietal fast spindles may be underrepresented, and we do not claim that it does. It does show, however, that the main hippocampal fMRI finding is not driven by idiosyncratic detections from one lateralised frontal electrode. We have made this clearer in the revised manuscript.

Finally, your concern about low thresholds is also important. As described in our response above, the revised analysis now defines SO and spindle thresholds from N2/3 sleep and applies these thresholds uniformly for descriptive comparisons across stages. We also tested the robustness of the hippocampal fMRI result across SO detection thresholds, and the effect remained significant across the 71st to 80th percentile range. We have therefore narrowed the interpretation in the revised manuscript. The main EEG-fMRI result reflects BOLD activity associated with frontal-channel-detected SO-spindle coupling during N2/3 sleep, rather than a full multichannel characterisation of all SO and spindle topographies.

Results, Page 12, Lines 247-250

“Third, to test whether the results depended on the use of a single lateralised frontal electrode, we repeated the EEG-informed fMRI GLM using events detected from Fz. Hippocampal activation during SO-spindle coupling again remained significant (*t*_(106)_ = 2.47, *p* = 0.0076), closely matching the original F3-based result (*t*_(106)_ = 2.50, *p* = 0.0070).”

Discussion, Page 18, Lines 380-394

“Third, sleep oscillation detection was based on a single frontal electrode. This choice improved signal stability and event timing in the prolonged simultaneous EEG-fMRI setting, but it did not exploit the full multichannel EEG information and cannot characterise the full spatial distribution of SOs and spindles. In particular, F3-based detection may be more sensitive to frontal SOs and frontal sigma activity than to the centro-parietal fast spindle component. We therefore interpret the EEG-informed fMRI results as reflecting BOLD activity associated with frontal-channel-detected SOs, spindles, and their coupling during N2/3 sleep. Future studies using multichannel or source-informed detection strategies, with separate treatment of slow and fast spindles, will be better suited to capture the spatial dynamics of these sleep oscillations. Fourth, the use of large anatomical ROIs may mask subregional contributions of specific thalamic nuclei or hippocampal subfields. Finally, without a memory task, we cannot establish a direct behavioral link between sleep-rhythm-locked activation and memory consolidation. Future studies combining ultra-high-field fMRI or iEEG with cognitive tasks, as well as multichannel or source-informed detection strategies that separately characterize slow and fast spindles, will be better suited to refine our understanding of subregional network dynamics and the functional significance of sleep oscillations.”

Methods, Page 25, Lines 556-566

“It is worth noting that the primary aim of EEG rhythm detection was to identify reliable event times for EEG-informed fMRI modelling. Detection was performed on the F3 electrode because this channel provided stable signal quality during prolonged nocturnal EEG-fMRI recordings. In our MR-compatible EEG setup, FCz was used as the online reference. Central electrodes close to this reference, and electrodes near the vertex that were in prolonged contact with the head coil during supine sleep, were more susceptible to reduced signal contrast, impedance drift, and pressure-related degradation of electrode-scalp contact. We therefore used F3 as a pragmatic choice to maximize reliable event timing in N2/3 sleep. This choice was not intended to characterise the full scalp topography of SOs or spindles, and it may underrepresent the centro-parietal fast spindle component. As a sensitivity analysis, we repeated the main EEG-informed fMRI GLM using Fz, a midline frontal electrode, with the same detection and modelling procedure.”

Re 7: It is not clear to me why/how larger voxels would reduce susceptibility-related distortions and partial volume effects. Usually, the opposite is true. This should be elaborated.

What we meant was that we chose a relatively large voxel size to preserve signal-to-noise ratio and whole-brain coverage within a feasible repetition time for a long overnight EEG-fMRI protocol. This choice is useful for maintaining BOLD sensitivity in sleep recordings, where head motion, physiological noise, and participant comfort are major practical constraints. We agree that it may not be accurate to describe it as reducing susceptibility-related distortion or partial volume effects.

We have rewritten the Methods to state this trade-off directly. The voxel size of 3.5 × 3.5 × 4.2 mm^3^ allowed whole-brain coverage with a TR of 2000 ms, which was important for modelling sleep-rhythm-related BOLD responses across the whole brain during prolonged nocturnal recordings. A smaller voxel size would have improved spatial specificity, but would also have required either a longer TR, reduced brain coverage, or lower SNR, none of which would have been ideal for the present EEG-fMRI sleep design. We now explicitly acknowledge the cost of this choice.

Methods, Page 21 Lines 453-463

“For the functional scans, whole-brain images were acquired using a T2*-weighted gradient echo-planar imaging (EPI) sequence sensitive to the BOLD contrast. The sequence parameters were as follows: 33 slices in interleaved ascending order, TR = 2000 ms, TE = 30 ms, voxel size = 3.5 × 3.5 × 4.2 mm^3^, FA = 90°, matrix = 64 × 64, gap = 0.7 mm. A relatively large voxel size was chosen to preserve signal-to-noise ratio while maintaining whole-brain coverage within a feasible repetition time. This compromise was important for the prolonged overnight EEG-fMRI sleep protocol, where head motion, physiological noise, participant comfort, and sustained acquisition stability are substantial practical constraints (Bodurka et al., 2007; Laufs et al., 2008). A smaller voxel size would have improved spatial specificity, but would have required either a longer repetition time, reduced brain coverage, or lower signal-to-noise ratio.”

**Reviewer #2 (Public review):**

In this study, Wang and colleagues aimed to explore brain-wide activation patterns associated with NREM sleep oscillations, including slow oscillations (SOs), spindles, and SO-spindle coupling events. Their findings reveal that SO-spindle events corresponded with increased activation in both the thalamus and hippocampus. Additionally, they observed that SO-spindle coupling was linked to heightened functional connectivity from the hippocampus to the thalamus, and from the thalamus to the medial prefrontal cortex-three key regions involved in memory consolidation and episodic memory processes.

This study's findings are timely and highly relevant to the field. The authors' extensive data collection, involving 107 participants sleeping in an fMRI while undergoing simultaneous EEG recording, deserves special recognition. If shared, this unique dataset could lead to further valuable insights.

Thank you for this encouraging assessment. We appreciate your recognition of the effort involved in collecting this simultaneous EEG-fMRI sleep dataset. Below, we respond directly to your remaining concern.

Comments on revisions:

The authors' efforts in revising the manuscript and addressing the reviewers' comments are certainly commendable. However, I remain concerned about potential issues in detecting sleep-related oscillations (SOs, spindles, and consequently coupled SO-spindle events), which may arise due to suboptimal parameter selection or inaccurate sleep staging, potentially impacting all subsequent analyses.

A review of Supplementary Tables 1-4 reveals an unusually high number of detected SOs and spindles during sleep stage N1 and REM sleep. While the authors correctly note that a percentile-based detection approach will always identify a certain number of events across sleep stages, the particularly high counts in N1 and REM are concerning. To mitigate the limitations of this method, the authors could have performed event detection independently of sleep stages (i.e., across the entire dataset for each participant) and subsequently assigned the detected events to the corresponding sleep stages. If the event counts in N1 and REM remained disproportionately high, this would indicate a fundamental issue with the detection procedure.

In the previous version, thresholds were estimated separately within each sleep stage. As you point out, this can force the detector to identify a relatively large number of SOs and spindles in N1 and REM, even when those waveforms should not be interpreted as canonical N2/3 events.

We have therefore revised the detection procedure so that event detection is no longer based on separate stage-wise thresholds. Following the logic of your suggestion, we first defined a fixed threshold for each participant and then assigned the detected events to their corresponding sleep stages afterwards. We used N2/3 sleep to define the SO and spindle thresholds because this is the stage in which these events are physiologically expected and most reliably observed. These same N2/3-derived thresholds were then applied unchanged to N1 and REM. This avoids the circularity of forcing a percentile-defined number of detections within each sleep stage.

With this revised procedure, detections outside N2/3 are clearly lower than those in N2/3. The mean densities are 2.95 SOs/min, 2.71 spindles/min, and 0.75 coupling events/min in N1, and 2.07 SOs/min, 1.81 spindles/min, and 0.43 coupling events/min in REM. We also agree with you that the remaining detections in N1 and REM should not be interpreted as physiological equivalents of canonical N2/3 SOs, spindles, or SO-spindle complexes. We now state this explicitly in the manuscript. They are reported only as descriptive detector outputs under the fixed N2/3-derived criterion. And we have revised all relevant sections of the manuscript, including “[Results, Page 6-7 Lines 134-148]; [Fig. 1e]; [Results, Page 9 Lines 175-191]; [Fig. 2b]; [Methods, Page 25-27, Lines 567-604]; [Fig. S2-S4]; [Table S2, S4].”

We also would like to clarify our sleep staging procedure. The sleep staging was first performed using an established automated algorithm, YASA toolkit (Vallat & Walker, 2021), and then manually reviewed by two sleep experts. More importantly for the central results, all EEG-informed fMRI GLM and PPI analyses were restricted to N2/3 sleep. Thus, N1 and REM detections did not enter the event regressors used for the main fMRI analyses and do not affect the interpretation of the hippocampal or thalamic findings.

Finally, we agree that the absolute number of SO-spindle coupling events depends on the detection threshold. We therefore tested whether the main fMRI result depended on the specific SO threshold. Hippocampal activation during SO-spindle coupling remained significant when the SO detection threshold was varied between the 71st and 80th percentiles, as shown in Fig. S6. We have made this clearer in the revised manuscript.

Results, Page 6-7 Lines 134-148

“Each sleep stage is characterised by distinct spectral properties and rhythmic waveforms, serving as physiological markers (Fig. 1c). Because SO and spindle detection relies on amplitude-based percentile thresholds, we avoided estimating separate thresholds within each sleep stage. Instead, for each participant, the SO and spindle thresholds were defined from N2/3 sleep only, where these rhythms are most abundant and physiologically expected, and the same fixed thresholds were then applied to N1 and REM for descriptive comparison.”

“Under this fixed N2/3-derived thresholding, detected SOs and spindles were larger and more frequent in N2/3 than in N1 or REM. SO and spindle amplitudes were significantly higher during N2/3 sleep (SO: 25.59 ± 1.49 μV; spindle: 7.39 ± 0.27 μV) than during N1 (SO: 20.15 ± 2.32 μV; spindle: 5.23 ± 0.27 μV) and REM sleep (SO: 19.84 ± 1.22 μV; spindle: 5.60 ± 0.22 μV; all *p* < 1e-4; Fig. 1e, Fig. S2). The corresponding event densities showed the same pattern, with 9.64 ± 0.25 SOs/min and 4.19 ± 0.10 spindles/min in N2/3, compared with 2.95 ± 0.16 SOs/min and 2.71 ± 0.14 spindles/min in N1, and 2.07 ± 0.17 SOs/min and 1.81 ± 0.14 spindles/min in REM (all *p* < 1e-4). We therefore report detections in N1 and REM only as descriptive outputs of the detector under a fixed N2/3-derived criterion, rather than as physiological equivalents of canonical N2/3 SOs or spindles.”

Fig. 1 legend, Page 8, Line 166-172

“e, Amplitudes (μV) of detected SOs (left) and spindles (right) across sleep stages. SO and spindle detection thresholds were defined from N2/3 sleep within each participant and then applied unchanged to N1 and REM for descriptive comparison. Detections in N1 and REM should therefore be interpreted as detector outputs under this fixed N2/3-derived criterion. The SO amplitudes were measured from the 0.16-1.25 Hz filtered EEG data, and spindle amplitudes were measured from the 12-16 Hz filtered EEG data. Each dot represents an individual participant. Error bars indicate SEM. *** *p* < 0.001.”

Results, Page 9 Lines 175-191

“SO-spindle coupling is considered important for sleep-dependent memory consolidation. In the current study, using the same N2/3-derived detection thresholds described above, we found that SO-spindle coupling occurred most frequently during N2/3 sleep (2.46 ± 0.06 events/min). Coupling density was significantly lower in N1 (0.75 ± 0.05 events/min, *t*_(106)_ = 23.54, *p* < 1e-4) and REM sleep (0.43 ± 0.04 events/min, *t*_(106)_ = 31.24, *p* < 1e-4; Fig. 2b, Table S2-S4), consistent with the expected predominance of SO-spindle coupling in NREM sleep (Ngo et al., 2013; Staresina et al., 2015). As with the individual SO and spindle detections, coupling events detected in N1 and REM were retained only for descriptive stage-wise reporting (see Table S2, S4). They were not used to support physiological claims about SO-spindle coupling in these stages, and they were not entered into the EEG-informed fMRI analyses. All subsequent fMRI GLM and PPI analyses were restricted to N2/3 sleep.”

“After extracting all N2/3 EEG epochs in which SO-spindle coupling occurred, we analysed their spectral and phase characteristics. The spindles were most likely to occur slightly before the UP-state peak of SOs (Fig. 2a, e), aligning with results from both animal studies (Maingret et al., 2016) and human research (Staresina et al., 2015). In our data, this pattern was consistent across subjects (Fig. 2d, Rayleigh test: *z* = 9.51, *p* < 1e-4), with the peak of the spindle aligned at an SO phase of −41.61 ± 0.86° (the SO UP-state peak is 0°).”

Fig. 2 legend, Page 10, Line 202-205

“b, SO-spindle coupling density across sleep stages, using SO and spindle detections obtained with fixed N2/3-derived thresholds. Coupling events in N1 and REM are shown only for descriptive comparison. The EEG-informed fMRI analyses used N2/3 coupling events only.”

Results, Page 11-12, Lines 242-247

“Second, because the absolute number of detected SO-spindle coupling events depends on the SO detection threshold, we examined whether the main EEG-fMRI results were sensitive to this parameter. To this end, we varied the SO percentile threshold and reconstructed the EEG-informed GLM at each level. Hippocampal activation during SO-spindle coupling remained significant across a range of thresholds (71st - 80th percentile; Fig. S6).”

Methods, Page 25-26, Lines 567-575

“Detection of SOs. Data were first bandpass-filtered between 0.16 and 1.25 Hz (Butterworth filter, order 3, bidirectional filtering for zero phase). After identifying all positive-to-negative zero crossings, potential SOs were defined based on the interval between consecutive zero crossings, ranging from 0.8 s to 3 s. For each potential SO, we calculated the amplitude range as the peak minus the trough. For each participant, the amplitude threshold was defined as the 75th percentile of candidate SO amplitude ranges observed during N2/3 sleep. This fixed N2/3-derived threshold was then applied unchanged across the recording for descriptive stage-wise summaries. Detected events were assigned to N1, N2/3 or REM according to the sleep-stage label at the event time. Only candidates exceeding this threshold were labelled as SOs, following previous work (Schreiner et al., 2021).”

Methods, Page 26, Lines 576-583

“Detection of sleep spindles. Detection of sleep spindles. Data were bandpass-filtered between 12 and 16 Hz (Butterworth filter, order 3, bidirectional filtering for zero phase). The root mean square (RMS) of the filtered signal was computed with a 200 ms sliding time window. For each participant, the spindle threshold was defined as the 75th percentile of RMS values observed during N2/3 sleep. This fixed N2/3-derived threshold was then applied unchanged across the recording for descriptive stage-wise summaries. Detected events were assigned to N1, N2/3 or REM according to the sleep-stage label at the event time. RMS segments exceeding this threshold for 0.5 s to 3 s were identified as spindles (Staresina et al., 2015).”

Methods, Page 26, Lines 584-591

“Detection of SO-spindle couplings. From the detected SOs and spindles, we identified the peak time of each spindle. Within each SO interval, we checked whether a spindle peak occurred; if so, that SO was labelled as an SO-spindle coupling event. For descriptive stage-wise summaries, coupling events were assigned to the sleep stage of the corresponding SO trough. For every SO-spindle coupling event, an epoch was created time-locked to the SO trough as the central reference, following Schreiner et al. (2021). We extracted data in a [−4 s to 4 s] window around this point, forming the epoch for each coupling event. For the EEG-informed fMRI analyses, only SO, spindle and SO-spindle coupling events detected during N2/3 sleep were used.”

Methods, Page 26-27, Lines 592-604

“The detection procedures described above were developed primarily for N2 and N3 sleep, where SOs, spindles and their coupling are physiologically expected and most reliably observed (Hahn et al., 2020; Helfrich et al., 2019; Helfrich et al., 2018; Ngo, Fell, & Staresina, 2020; Schreiner et al., 2022; Schreiner et al., 2021; Staresina et al., 2015; Staresina et al., 2023). Because percentile-based thresholds can otherwise force the detector to label events in every sleep stage, we did not estimate separate thresholds within N1 or REM. Instead, for each participant, all SO and spindle thresholds were defined from N2/3 sleep and then applied uniformly across the recording. Tables S1 and S3 report detailed statistical information on sleep rhythm and N2/3 events detection. The N1 and REM events detection reported in Tables S2 and S4, and illustrated in Fig. S2-S4, should therefore be interpreted as descriptive detector outputs under this fixed N2/3-derived criterion, rather than as evidence for canonical N2/3 SOs, spindles or physiological SO-spindle complexes in those stages. These detections were not used in the EEG-informed fMRI GLM or PPI analyses, which were restricted to N2/3 sleep.”

**Reviewer #3 (Public review):**

Summary:

Wang et al., examined the brain activity patterns during sleep, especially when locked to those canonical sleep rhythms such as SO, spindle, and their coupling. Analyzing data from a large sample, the authors found significant coupling between spindles and SOs, particularly during the up-state of the SO. Moreover, the authors examined the patterns of whole-brain activity locked to these sleep rhythms. The authors next investigated the functional connectivity analyses, and found enhanced connectivity between the hippocampus and the thalamus and the medial PFC. These results reinforced the theoretical model of sleep-dependent memory consolidation, such that SO-spindle coupling is conducive for systems-level memory reactivation and consolidation.

Strengths:

There are obvious strengths in this work, including the large sample size, state-of-the-art neuroimaging and neural oscillation analyses, and the richness of results. The results now inform hemodynamic neural activity that coincided with SO-spindle couplings.

Weaknesses:

My earlier comments were about the inability to make inferences on memory given the lack of memory tasks, and the weakness in using the open-ended cognitive state decoding.

Comments on revisions:

The current revision has addressed these major concerns. The authors expanded discussions regarding the theoretical implications of the work in a more nuanced manner.

Thank you for taking the time to re-evaluate the manuscript. We are pleased that the revised Discussion now reads as more nuanced, especially in relation to the limits of the memory-related interpretation. Your earlier comments helped us sharpen both the claims and the framing, and we are grateful for that.

References:

Bergmann, T. O., Mölle, M., Diedrichs, J., Born, J., & Siebner, H. R. (2012). Sleep spindle-related reactivation of category-specific cortical regions after learning face-scene associations. Neuroimage, 59(3), 2733-2742.

Bodurka, J., Ye, F., Petridou, N., Murphy, K., & Bandettini, P. A. (2007). Mapping the MRI voxel volume in which thermal noise matches physiological noise—implications for fMRI. Neuroimage, 34(2), 542-549.

Caporro, M., Haneef, Z., Yeh, H. J., Lenartowicz, A., Buttinelli, C., Parvizi, J., & Stern, J. M. (2012). Functional MRI of sleep spindles and K-complexes. Clinical neurophysiology, 123(2), 303-309.

Czisch, M., Wehrle, R., Stiegler, A., Peters, H., Andrade, K., Holsboer, F., & Sämann, P. G. (2009). Acoustic oddball during NREM sleep: a combined EEG/fMRI study. PloS one, 4(8), e6749.

Fogel, S., Albouy, G., King, B. R., Lungu, O., Vien, C., Bore, A., Pinsard, B., Benali, H., Carrier, J., & Doyon, J. (2017). Reactivation or transformation? Motor memory consolidation associated with cerebral activation time-locked to sleep spindles. PloS one, 12(4), e0174755.

Hahn, M. A., Heib, D., Schabus, M., Hoedlmoser, K., & Helfrich, R. F. (2020). Slow oscillation-spindle coupling predicts enhanced memory formation from childhood to adolescence. Elife, 9, e53730.

Hale, J. R., White, T. P., Mayhew, S. D., Wilson, R. S., Rollings, D. T., Khalsa, S., Arvanitis, T. N., & Bagshaw, A. P. (2016). Altered thalamocortical and intra-thalamic functional connectivity during light sleep compared with wake. Neuroimage, 125, 657-667.

Helfrich, R. F., Lendner, J. D., Mander, B. A., Guillen, H., Paff, M., Mnatsakanyan, L., Vadera, S., Walker, M. P., Lin, J. J., & Knight, R. T. (2019). Bidirectional prefrontal-hippocampal dynamics organize information transfer during sleep in humans. Nature Communications, 10(1), 3572.

Helfrich, R. F., Mander, B. A., Jagust, W. J., Knight, R. T., & Walker, M. P. (2018). Old brains come uncoupled in sleep: slow wave-spindle synchrony, brain atrophy, and forgetting. Neuron, 97(1), 221-230. e224.

Huang, Q., Xiao, Z., Yu, Q., Luo, Y., Xu, J., Qu, Y., Dolan, R., Behrens, T., & Liu, Y. (2024). Replay-triggered brain-wide activation in humans. Nature Communications, 15(1), 7185.

Ilhan-Bayrakcı, M., Cabral-Calderin, Y., Bergmann, T. O., Tüscher, O., & Stroh, A. (2022). Individual slow wave events give rise to macroscopic fMRI signatures and drive the strength of the BOLD signal in human resting-state EEG-fMRI recordings. Cerebral Cortex, 32(21), 4782-4796.

Laufs, H., Daunizeau, J., Carmichael, D. W., & Kleinschmidt, A. (2008). Recent advances in recording electrophysiological data simultaneously with magnetic resonance imaging. Neuroimage, 40(2), 515-528.

Maingret, N., Girardeau, G., Todorova, R., Goutierre, M., & Zugaro, M. (2016). Hippocampo-cortical coupling mediates memory consolidation during sleep. Nature Neuroscience, 19(7), 959-964.

Moehlman, T. M., de Zwart, J. A., Chappel-Farley, M. G., Liu, X., McClain, I. B., Chang, C., Mandelkow, H., Özbay, P. S., Johnson, N. L., & Bieber, R. E. (2019). All-night functional magnetic resonance imaging sleep studies. Journal of neuroscience methods, 316, 83-98.

Ngo, H.-V., Fell, J., & Staresina, B. (2020). Sleep spindles mediate hippocampal-neocortical coupling during long-duration ripples. Elife, 9, e57011.

Ngo, H. V., Martinetz, T., Born, J., & Molle, M. (2013). Auditory closed-loop stimulation of the sleep slow oscillation enhances memory. Neuron, 78(3), 545-553.

Picchioni, D., Horovitz, S. G., Fukunaga, M., Carr, W. S., Meltzer, J. A., Balkin, T. J., Duyn, J. H., & Braun, A. R. (2011). Infraslow EEG oscillations organize large-scale cortical–subcortical interactions during sleep: a combined EEG/fMRI study. Brain research, 1374, 63-72.

Schabus, M., Dang-Vu, T. T., Albouy, G., Balteau, E., Boly, M., Carrier, J., Darsaud, A., Degueldre, C., Desseilles, M., & Gais, S. (2007). Hemodynamic cerebral correlates of sleep spindles during human non-rapid eye movement sleep. Proceedings of the National Academy of Sciences, 104(32), 13164-13169.

Schreiner, T., Kaufmann, E., Noachtar, S., Mehrkens, J.-H., & Staudigl, T. (2022). The human thalamus orchestrates neocortical oscillations during NREM sleep. Nature Communications, 13(1), 5231.

Schreiner, T., Petzka, M., Staudigl, T., & Staresina, B. P. (2021). Endogenous memory reactivation during sleep in humans is clocked by slow oscillation-spindle complexes. Nature Communications, 12(1), 3112.

Staresina, B. P., Bergmann, T. O., Bonnefond, M., van der Meij, R., Jensen, O., Deuker, L., Elger, C. E., Axmacher, N., & Fell, J. (2015). Hierarchical nesting of slow oscillations, spindles and ripples in the human hippocampus during sleep. Nature Neuroscience, 18(11), 1679-1686.

Staresina, B. P., Niediek, J., Borger, V., Surges, R., & Mormann, F. (2023). How coupled slow oscillations, spindles and ripples coordinate neuronal processing and communication during human sleep. Nature Neuroscience, 1-9.

Vallat, R., & Walker, M. P. (2021). An open-source, high-performance tool for automated sleep staging. Elife, 10.
